# Herbicide Efficacy of Spot Spraying Systems in Fallow and Postharvest in the Pacific Northwest Dryland Wheat Production Region

**DOI:** 10.3390/plants10122725

**Published:** 2021-12-11

**Authors:** Nicholas G. Genna, Jennifer A. Gourlie, Judit Barroso

**Affiliations:** Columbia Basin Agricultural Research Center, Oregon State University, 48037 Tubbs Ranch Road, Adams, OR 97810, USA; nicholas.genna@usda.gov (N.G.G.); jennifer.gourlie@oregonstate.edu (J.A.G.)

**Keywords:** no-till, precision agriculture, WEED-IT, WeedSeeker

## Abstract

Real-time spot spraying technology has the potential to reduce herbicide costs and slow herbicide resistance. However, few studies exist on the efficacy of this technology in the Pacific Northwest (PNW). This research compared the herbicide efficacy (reduction in weed density and cover) of WEED-IT and WeedSeeker spot spraying systems to uniform spraying in fallow and postharvest in 2019 and 2020. Weed community types included naturally occurring weeds, natural + Russian thistle (*Salsola tragus* L.), or natural + kochia (*Bassia scoparia* (L.) A. J. Scott). Herbicides included glyphosate or the pre-mix bromoxynil + pyrasulfotole. Additionally, herbicide efficacy was studied with short stubble (~10 cm), tall stubble (~25 cm), and normal stubble (~20 cm) with chaff and straw removed. In fallow, herbicide efficacy was 1.5 times higher for uniform applications than for WEED-IT or WeedSeeker in 2019 and 2020. Herbicide efficacy was also 1.9 times higher for uniform applications in postharvest in 2019 but no differences were found in 2020. The weed community impacted herbicide efficacy but herbicide efficacy did not differ between residue management treatments. Finally, WEED-IT and WeedSeeker used 53% less herbicide volume in comparison to uniform applications. This research demonstrated that spot spraying technology can be efficacious and economical for growers in the PNW.

## 1. Introduction

A no-till wheat-fallow rotation relies on herbicides to control weeds in fallow and postharvest. Herbicides are traditionally applied uniformly across fields regardless of weed cover or density. Timmermann et al. [1] demonstrated that weed mapping could reduce herbicide costs by 54% compared to a uniform application, but higher labor costs prohibit manual weed mapping by growers. Real-time spot spraying technology was developed to overcome manual labor requirements by differentiating weeds from soil without the need of a weed map [2,3]. Light activated sensors turn individual spray nozzles on and off when weeds are detected to minimize coverage of weed-free soil and maximize herbicide savings. WEED-IT^®^ (Rometron B.V., Steenderen, The Netherlands) and WeedSeeker^®^ (Trimble, Sunnyvale, CA, USA) are two spot spraying systems marketed to farmers with the potential of reducing herbicide costs and slowing herbicide resistance. However, their efficacy is unknown in fallow and postharvest in the wheat farming region of the inland Pacific Northwest (PNW). 

Glyphosate is the primary non-selective herbicide used in fallow and postharvest in the PNW. Glyphosate use increased dramatically after resistant crops were developed in 1996, but the current utility of glyphosate is waning with the concomitant global increase in glyphosate resistant weeds [4,5]. Decades of glyphosate use in this region has selected for resistant genotypes of major agronomic weeds including Russian thistle (*Salsola tragus* L.) and downy brome (*Bromus tectorum* L.) [6,7,8]. Weeds in this region are also resistant to other commonly used herbicides including chlorsulfuron, metsulfuron-methyl, or 2,4-D [9]. A concern of rapidly developing and spreading herbicide resistance is returning farmers to tillage that is threatening soil conservation gains made with no-till farming practices [10]. Growers continuing with no-till understand the need to alternate or tank-mix products with glyphosate to slow resistance, but alternatives to glyphosate are cost prohibitive. Reduced herbicide costs and the potential to afford herbicides with different modes of action is motivating farmers to adopt spot spraying technology [11]. For instance, spot spraying technology has been shown to use 20–60% less herbicide volume than a broadcast application in the PNW [12,13,14]. With those savings, farmers could afford herbicides that are more expensive than glyphosate for an herbicide resistance management strategy [15]. 

WeedSeeker and WEED-IT work by emitting light in the visible spectrum and detecting returning chlorophyll fluorescence in the near infrared spectrum [16]. The more recently developed WEED-IT system possesses some advantages over the original WeedSeeker system, such as automatic calibration, spray pattern adjustments, and sensitivity adjustments. Nevertheless, newly emerging weeds may escape detection by both systems while mature weeds may not receive full coverage if a spot sprayer is not calibrated properly [17,18]. Differing environmental conditions such as cloud cover and light intensity can also impact spot sprayer efficacy [19]. Insufficient herbicide coverage and suboptimal weed detection can limit the efficacy of spot spraying systems and potentially detract farmers from the technology. To improve the efficacy of spot sprayers, growers may choose to use higher herbicide rates since the effective volume applied to a given field may be less than a uniform application at the label rate; albeit negating some cost savings [13]. 

To encourage adoption of this technology, growers must understand the relative efficacy of spot spraying systems in differing conditions such as with small seedlings, larger weeds, and the impact of plant debris such as chaff, straw, and stubble [19]. Three trials were conducted in 2019 and 2020 to address these issues by comparing WEED-IT and WeedSeeker spot spraying systems to uniform spraying in fallow, postharvest, and by differing residue management in fallow and postharvest. 

## 2. Materials and Methods

### 2.1. Location

Experiments were established in Umatilla County at the Columbia Basin Agricultural Research Center (CBARC) in Adams, Oregon (45°43′10.41” N; 118°37′37.09” W) during 2019 and 2020. CBARC is rain-fed with a long-term average precipitation of 421 mm yr^−1^. The soil is a Walla Walla silt loam (8% clay, 27% sand, 65% silt) with 2.3% organic matter and a pH of 5.4. The fallow and postharvest trials were located within fields managed conventionally while the residue trial was located within a no-till field. The fallow and postharvest trials were repeated during two consecutive years in different fields while the residue trial was conducted in the same field for two years.

### 2.2. Experimental Trials and Design

Fallow. The fallow trial was a split-plot randomized complete block design with four replications. Spray system was the main plot factor and infestation type was the sub-plot factor. Main plots were 3.0 × 9.1 m and sub-plots were 3.0 × 3.0 m. Spray systems included a continuous broadcast application (denoted uniform), WEED-IT, and WeedSeeker. WeedSeeker was not operational for the fallow 2019 trial but was used for all other trials in 2019 and 2020. Infestation types included naturally occurring weeds, natural + seeded Russian thistle, and natural + seeded kochia. Each infestation is hereafter referred to as natural, Russian thistle, and kochia, respectively. Russian thistle and kochia were chosen due to their prevalence in the region and difficulty to control with typical broadcast herbicide applications. Russian thistle and kochia seed were spread by hand at a rate of 0.43 g m^−2^ and 1.08 g m^−2^, respectively, and worked into the upper 5 cm of soil with a Turbo-Max vertical tillage implement (Great Plains Manufacturing, Salina, KS, USA) on 29 April 2019 and 24 March 2020. Seeding densities for Russian thistle and kochia were chosen based on prior experience that would achieve a typical weed infestation in this region. The experimental design was duplicated across glyphosate (GlyStar^®^ Plus, Albaugh, Ankeny, IA, USA) and bromoxynil + pyrasulfotole (Huskie^®^, Bayer CropScience, Research Triangle Park, NC, USA) herbicides, with the glyphosate herbicide changed in 2020 to a higher concentration product (GlyStar^®^ 5 Extra, Albaugh, Ankeny, IA, USA) (Table 1). Herbicide was applied on 8–10 July 2019 and 7–11 May 2020. The fallow site location and prior field conditions differed each year with wheat preceding the 2019 site and fallow preceding the 2020 site. 

Postharvest. The postharvest trial was a split-plot randomized complete block design with four replications. Spray system was the main plot factor and infestation type was the sub-plot factor. Main plots were 3.0 × 9.1 m and subplots were 3.0 × 3.0 m. Spray systems included uniform, WEED-IT, and WeedSeeker. Infestation types included natural, natural + Russian thistle, and natural + kochia. Russian thistle and kochia seed was spread by hand at a rate of 0.43 g m^−2^ and 1.08 g m^−2^, respectively, on 17 April 2019 and 13 March 2020. Weed seeds were worked into the soil by subsequently seeding spring wheat (‘WB6341′) 2.5 cm deep in 25 cm rows at 100 kg ha^−1^ on 17 April 2019 with a no-till drill (Great Plains Manufacturing, Salina, KS, USA). Spring wheat (‘Ryan’) was seeded 1.3 cm deep in 19 cm rows at 123 kg ha^−1^ on 16 March 2020 with a Case (Racine, WI, USA) 5300 double disk conventional drill. The crop was harvested on 13 August 2019 and 5 August 2020. Herbicide applications were made on 26–28 August 2019 and 11–12 August 2020. The experimental design was duplicated across glyphosate and bromoxynil + pyrasulfotole herbicides with the glyphosate herbicide changed in 2020 (Table 1). The postharvest site location differed each year with both sites following wheat.

Residue. The residue trial was a split-plot randomized complete block design with four replications. Stubble height was the main plot factor and spray system was the sub-plot factor. Main plots were 9.1 × 45.7 m and sub-plots were 9.1 × 15.2 m. Stubble height treatments included short (~10 cm), tall (~25 cm), and regular (~20 cm). Residue was removed from the regular stubble treatment by affixing a tarp to the rear of a combine. Spray systems included uniform, WEED-IT, and WeedSeeker. The residue trail was a two-year experiment located in the same location in 2019 and 2020. Therefore, the residue trial was postharvest in 2019 and fallow in 2020. Spring wheat (‘WB6341′) was seeded 2.5 cm deep in 25 cm rows at 95 kg ha^−1^ on 10 April 2019 with a no-till drill (Great Plains Manufacturing, Salina, KS, USA). The crop was harvested on 14 August 2019. Bromoxynil + 2,4-D (Deadbolt^®^, Wilbur-Ellis, San Francisco, CA, USA) was applied postharvest on 27–30 August 2019. Glyphosate mixed with bromoxynil + pyrasulfotole was applied in fallow on 20 April–1 May 2020 (Table 1).

### 2.3. Sprayer Information

Each spray system was calibrated to deliver the same herbicide rate. WeedSeeker (model 650) operated at 45–50 psi with a flow rate of 192 L ha^−1^ through TeeJet (TeeJet Technologies, Springfield, IL, USA) TP6502 flat fan nozzles positioned 51 cm above the ground and spaced 30 cm apart on a 3 m boom. WEED-IT (generation 2) in 2019 operated at 45 psi with a flow rate of 442 L ha^−1^ in the fallow trial and 519 L ha^−1^ in the postharvest and residue trials. WEED-IT utilized Agrotop (Timmorim, Israel) Spotfan 40-03 flat fan nozzles spaced 20 cm apart and positioned 69 cm above the ground on a 3 m boom. WEED-IT was recalibrated in 2020 for larger plants by changing the nozzles to Albuz (Évreux, France) TF 60-04 producing a cone spray. WEED-IT treatments in 2020 were mixed assuming a spray volume of 140 L ha^−1^ but were unintentionally sprayed at 468 L ha^−1^ (3× the intended herbicide rate) (Table 1). WeedSeeker and WEED-IT were pulled by the same tractor at 7 km h^−1^ in 2019 and 2020. A CO_2_ powered backpack sprayer was used to spray the uniform treatments for the fallow and postharvest trials in 2019 and 2020. Spray volume was 140 L ha^−1^ through TeeJet XR80015VS nozzles positioned 46 cm above the ground and spaced 46 cm apart on a 2.75 m boom. The uniform treatment in the residue trial in 2019 and 2020 was sprayed with a 9.1 m boom mounted sprayer pulled by a tractor at 7 km h^−1^ with a spray volume of 140 L ha^−1^ through TeeJet AIC11003 nozzles positioned 61 cm above the ground and spaced 51 cm apart. 

### 2.4. Data Collection and Statistical Analyses

Weed density counts and weed cover was estimated for all species within each sub-plot by placing a 0.5 m^2^ sampling frame in four-six random locations. Evaluations were made before herbicide applications (denoted pre-spray) and at three and six weeks after treatment (denoted post-spray). Density counts and cover estimations were transformed to herbicide efficacy with the formula:

$$\mathrm{Herbicide}\;\mathrm{efficacy}=\frac{\left(\mathrm{pre-spray}\;\mathrm{evaluation}-\mathrm{post-spray}\;\mathrm{evaluation}\right)}{\mathrm{pre-spray}\;\mathrm{evaluation}}\times 100$$

Three and six week post-spray evaluations were averaged before analyses. Negative efficacy values were made to equal zero since herbicide control was of primary interest. Data were subjected to the non-parametric Kruskal–Wallis one-way analyses of variance due to skewing that could not be accommodated with transformation. The homogeneity of variance assumption was assessed with the Fligner-Killeen test. Dunn’s test with the Bonferroni adjustment was used for means separation at α = 0.05. Dunn’s test without the Bonferroni adjustment was used when post-hoc tests were not significant with the Bonferroni adjustment. Data that violated the constant variance assumption was analyzed with individual two-sample Welch *t*-tests. Fallow, postharvest, and residue trials were analyzed independently. Years and herbicides were analyzed independently for each trial. RStudio v.1.2 software (RStudio Team, Boston, MA, USA) was used to analyze and visualize all data.

## 3. Results and Discussion

### 3.1. Fallow

Pre-spray weed density averaged 32 plants m^−2^ and cover averaged 31% across all plots in 2019. The most abundant weed species by cover in natural plots before herbicide applications were prostrate pigweed (*Amaranthus albus* L., 10%), cutleaf nightshade (*Solanum triflorum* Nutt., 4%), and prickly lettuce (*Lactuca serriola* L., 2%). Russian thistle was the most abundant weed by cover in Russian thistle plots at 34% followed by prostrate pigweed at 6%. Prostrate pigweed was the most abundant species in kochia plots at 9% followed by kochia at 7%. Density and cover were significantly affected by spray system, with uniform applications providing 1.4 times greater control than precision applications with respect to density. Density and cover were also affected by weed community, with glyphosate providing the greatest reduction in natural plots, and bromoxynil + pyrasulfotole in Russian thistle plots. The latter is due to Russian thistle being easier to control with bromoxynil + pyrasulfotole than with glyphosate [7]. The spray system × weed community interaction was significant for density and cover with glyphosate but only for density with bromoxynil + pyrasulfotole. Glyphosate efficacy was greatest in uniformly sprayed kochia plots while the lowest efficacy was found in Russian thistle plots sprayed with WEED-IT. With bromoxynil + pyrasulfotole, the greatest control was found in Russian thistle plots sprayed uniformly while the lowest control was found in natural and kochia plots sprayed with WEED-IT (Table 2).

The 2020 fallow trial was sprayed two months earlier than in 2019. Emerging weeds were more numerous at 47 plants m^−2^ while cover was lower at 11% across all plots. Pre-spray cover in natural plots was dominated by prickly lettuce at 9% and cutleaf nightshade at 2%. Prickly lettuce was the most abundant weed in Russian thistle plots at 7% followed by Russian thistle at 5%. Kochia plots were dominated by prickly lettuce at 9% while kochia cover was <1%. As in 2019, spray system affected density and cover with bromoxynil + pyrasulfotole while only cover differed with glyphosate. Uniform applications provided 1.5 times higher herbicide efficacy than precision spraying systems with respect to density. However, weed community did not affect herbicide efficacy for either herbicide in 2020. The spray system × weed community interaction was only significant for weed cover with both herbicides. Uniform glyphosate applications in the Russian thistle weed community provided 30% control while WEED-IT or WeedSeeker provided 0% control in natural, kochia, or Russian thistle plots. Similar interactions were found with bromoxynil + pyrasulfotole for cover (Table 2).

The fallow trial demonstrated that a uniform application can provide greater herbicide efficacy when compared to a spot sprayer applying the same herbicide rate. In contrast, Riar et al. [20] observed similar control with broadcast and spot-spraying treatments applying the same or higher glyphosate rates in fallow. Weeds were large in 2019 due to a late spray application. Spot sprayers likely provided insufficient coverage of larger weeds that was better achieved with a uniform application. Larger weeds may be insufficiently covered if the spot sprayer is not calibrated properly (i.e., does not turn on early enough or continue spraying long enough to provide complete coverage) or if spray nozzles are inappropriate for larger weeds. Uniform spraying also outperformed WEED-IT in 2020 when this spray system was applying a 3× rate of glyphosate. Prickly lettuce was the predominant weed in 2020, which glyphosate provided a maximum control efficacy of only 43.5% when uniformly sprayed. Bromoxynil + pyrasulfotole provided superior weed control and there was no significant difference between uniform and WEED-IT treatments with this herbicide. The reduced performance of WeedSeeker in 2020 was most likely due to insufficient detection of smaller weeds. Young et al. [14] also found reduced detection of Russian thistle plants with WeedSeeker (model 600) when plants were shorter than 8 cm tall and smaller than 4 cm in diameter. McCarthy [19] compared WEED-IT and WeedSeeker directly in fallow and demonstrated that WeedSeeker failed to detect smaller weeds more often than WEED-IT. However, the next generation WeedSeeker 2 should provide superior performance over the prior model tested here. 

### 3.2. Postharvest

Initial weed density and cover across all plots in 2019 were 13 plants m^−2^ and 20%, respectively. The most abundant weed species by cover in natural plots were Russian thistle at 4% and prickly lettuce at 1%. In Russian thistle plots, Russian thistle cover was highest at 44% followed by prickly lettuce at <1%. Kochia was the most abundant weed in Kochia plots at 5%, followed by Russian thistle at 3%, and prickly lettuce at 1%. As in the fallow trial, uniform spraying reduced weed density and cover by 1.9 times more than precision spraying systems across herbicides. Herbicide efficacy was also affected by weed community for both herbicides with the greatest control found in Russian thistle communities and the lowest in natural plots. The spray system × weed community interaction was significant for density and cover with glyphosate but only for cover with bromoxynil + pyrasulfotole. The greatest efficacy of both herbicides was found in Russian thistle plots sprayed uniformly (Table 3).

Initial weed density and cover was lower in 2020 across all plots at 9 plants m^−2^ and 5.5%, respectively. The most abundant species by cover in natural plots before herbicide applications were Russian thistle at 1% and <1% contribution by prickly lettuce and common lambsquarters (*Chenopodium album* L.). Russian thistle was the most abundant weed in Russian thistle plots at 11% with <1% of common lambsquarters. Russian thistle was also the most abundant in kochia plots at 2% followed by kochia at <1%. Herbicide efficacy was lower in 2020 with respect to plant density with glyphosate control decreasing from 35% to 13% and bromoxynil + pyrasulfotole control decreasing from 44% to 4%. In contrast to 2019, weed cover was only affected by spray system with glyphosate with the lowest efficacy provided by WeedSeeker. Similar to 2019, weed community affected herbicide efficacy with the greatest efficacy found in Russian thistle plots. The spray system × weed community interaction was significant for density and cover for both herbicides. For glyphosate, herbicide efficacy was highest in Russian thistle plots sprayed by WEED-IT (3× higher rate for WEED-IT in 2020) while the greatest control with bromoxynil + pyrasulfotole was found in Russian thistle plots sprayed uniformly (Table 3). Herbicide efficacy was similar between spray systems within the Russian thistle community for both herbicides. Young et al. [14] reached a similar conclusion demonstrating that herbicide efficacy was similar between a broadcast sprayer and a spot sprayer in postharvest with Russian thistle across two years. Higher herbicide efficacy in the Russian thistle community indicates that this species was the easiest to control with the herbicides used in this trial. 

Overall, herbicide efficacy was two times lower in the postharvest trials in comparison to the fallow trials for uniform and spot spraying, supporting the hypothesis that drier conditions, larger weeds, and the abundance of stubble and other plant debris reduces herbicide control. However, the reduction in efficacy was similar between uniform and precision sprayers, indicating that the optical sensors were not significantly impacted by the presence of crop residue. 

### 3.3. Residue

In 2019, pre-spray weed cover averaged 12% across all plots in postharvest. Russian thistle dominated the weed community at 10% with 1% of prickly lettuce intermixed. Uniform spraying provided the greatest reduction of cover at 77%, while herbicide efficacy of precision sprayers was only 32% on average. Residue management did not affect herbicide efficacy while the spray system × residue management interaction was significant. All treatment combinations provided statistically similar herbicide efficacy except for the short stubble treatment sprayed by WEED-IT which provided the lowest efficacy (Table 4). 

Cover was higher in 2020 in fallow averaging 49%, with tumble mustard (*Sisymbrium altissimum* L.) being the most abundant species at 22% followed by downy brome at 18% and prickly lettuce at 6%. Herbicide efficacy was affected by the spray system but not by the residue management of the previous year. WEED-IT provided the greatest reduction in weed cover at 95% while WeedSeeker provided the lowest efficacy at 88%. The spray system × residue management interaction was significant with uniform and WEED-IT treatments providing the greatest efficacy and the WeedSeeker treatment without residue (tarp treatment) providing the lowest efficacy (Table 4).

Varying stubble height or collecting chaff and straw did not influence herbicide efficacy in this research. In contrast, Simao et al. [21] found taller 70 cm stubble intercepted 37% of applied herbicide while shorter 30–35 cm stubble intercepted only 23%. Similarly, Ghadiri et al. [22] showed that 60% of applied atrazine was retained by wheat stubble after application, demonstrating that stubble can reduce herbicide coverage. Together, work by Simao et al. [21] and Ghadiri et al. [22] suggests that taller stubble should reduce herbicide efficacy in comparison to shorter stubble. However, the maximum stubble height that could be achieved in 2019 was ~25 cm due to a weak spring crop, likely driving the absence of a treatment effect in our research. Consequently, thin stubble shorter than 25 cm should not concern growers while reduced herbicide efficacy may be possible with taller or thicker stubble. 

The herbicide efficacy of WEED-IT was similar to the uniform treatment in 2020 for most of the trials, potentially due to the 3× herbicide rate. Fischer et al. [13] also observed greater control of Rush skeletonweed (*Chondrilla juncea* L.) with WEED-IT using double the label rate. Only uniform glyphosate treatments in the fallow trial provided greater control than WEED-IT, but that was likely driven by the resistance of prickly lettuce to glyphosate which has been reported in this region [23]. Greater herbicide coverage from a uniform application may therefore be more important when herbicide efficacy is low. The lower control of prickly lettuce with WEED-IT was also likely not a detection problem since bromoxynil + pyrasulfotole should have demonstrated a similar pattern. 

### 3.4. Herbicide Savings with Spot Sprayers

Spot spraying systems applied 53% less herbicide volume on average in comparison to their continuous spray rate (Table 5). Findings from this study support previous research in the PNW suggesting that spot sprayers can reduce herbicide costs by approximately half [12,13,14]. However, WEED-IT’s effective spray volume averaged 281 L ha^−1^, often exceeding the 140 L ha^−1^ applied in uniform treatments due to a high continuous spray volume of 494 L ha^−1^ on average. In contrast, WeedSeeker’s effective spray volume was considerably lower at 74 L ha^−1^ (Table 5). This highlights that the output volume of a spot sprayer must be similar to a uniform sprayer to ensure that the effective spray volume is lower than a uniform herbicide application. 

**Figure 1:**
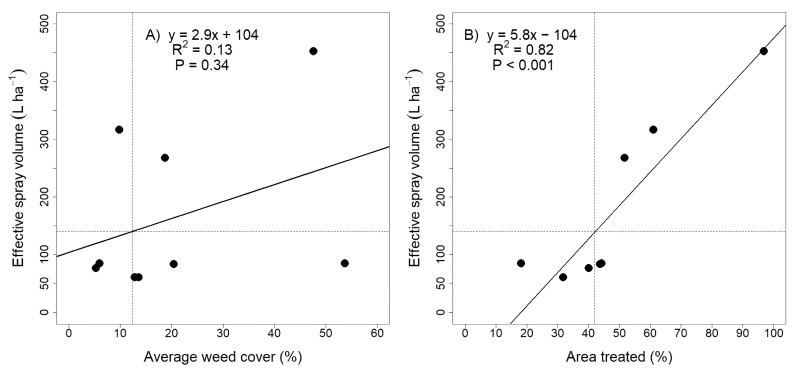
Relationship between (**A**) average weed cover (%) and effective spray volume (L ha^−1^) or (**B**) area treated (%) and effective spray volume (L ha^−1^) for all trials and sprayers in 2019 and 2020. Effective spray volume is the output volume of a spot sprayer in spot spraying mode. Area treated is the effective spray volume divided by the continuous spray volume (Table 5).

Fischer et al. [13] conclude that spot sprayers may be economical if weed cover is less than 30%. However, the 30% conclusion is derived from area treated and not cover. In this research, weed cover provided a poor relationship with effective spot sprayer output volume (Figure 1A). That relationship improved dramatically when weed cover was replaced with area treated (Figure 1B). In this research, an effective spot spray output volume, equivalent to the uniform treatment at 140 L ha^−1^, was achieved when the area treated by spot sprayers was approximately 42% or less. This translates to a weed cover of approximately 12%, significantly less than recommended by Fischer et al. [13]. The improved relationship with area treated also demonstrates that area treated should be used preferentially over weed cover when assessing the economics of spot sprayers.

Any conclusion about maximum weed cover in relation to spot sprayer economics is also dependent on the continuous spray volume of a specific spot sprayer, which may differ from the 275 L ha^−1^ continuous spot sprayer volume used by Fischer et al. [13]. For example, using the 30% threshold proposed by Fischer et al. [13], plant cover would have to be less than 30% for a spot sprayer with a continuous spray volume higher than 275 L ha^−1^. Similarly, the plant cover threshold would be higher than 30% if the spot sprayer’s volume was lower than 275 L ha^−1^.

If a spot sprayer’s effective spray volume depends on (1) area treated by a spot sprayer in spot spray mode and (2) the continuous spraying volume of a given spot sprayer, then a dedicated study is needed to provide a rapidly accessible table for growers to assess when a spot sprayer is no longer economical given a particular field’s weed cover and a spot sprayers continuous spraying volume. Similarly, manufacturers should develop technology that provides a real-time assessment of area treated as growers are applying herbicide. This could provide growers the flexibility to turn off a spot sprayer when weed cover is high in favor of a broadcast application.

## 4. Conclusions

Weeds at either end of the size spectrum, either small or large, may be an issue for spot sprayers in fallow in the PNW and higher herbicide rates alone may not overcome issues related to suboptimal weed detection. Higher herbicide rates can overcome issues related to sub-optimal coverage, which is most probable in postharvest applications when weeds are large and mature. Choosing herbicide products or tank-mixes that are best for a predominant species or weed community will further maximize control with spot sprayers. Despite the greater efficacy of uniform applications in this research, significant herbicide savings provided by spot sprayers should encourage growers to adopt this technology.

## Figures and Tables

**Table 1:** Herbicide tank mixtures and herbicide rates for fallow, postharvest, and residue trials in 2019 and 2020.

Year	Trial	Active Ingredient	Herbicide Rate ^a^	Tank Mixture	Delivered Rate ^a^		
					Uniform	WEED-IT	WeedSeeker
2019	Fallow	Glyphosate	833 g a.e. ha^−1^	Glystar Plus + 20 g Ammonium Sulfate (AMS) L^−1^	1×	1×	1×
		Bromoxynil + Pyrasulfotole	230 g a.i. ha^−1^ + 40 g a.i. ha^−1^	Huskie + 157 g a.i. ha^−1^ Section 3 (clethodim) + 2.3 L Crop oil concentrate (COC) ha^−1^ + 9.4 L Solution 32 ha^−1^	1×	1×	1×
	Postharvest	Glyphosate	833 g a.e. ha^−1^	Glystar Plus + 20 g AMS L^−1^	1×	1×	1×
		Bromoxynil + Pyrasulfotole	230 g a.i. ha^−1^ + 40 g a.i. ha^−1^	Huskie + 157 g a.i. ha^−1^ Section 3 (clethodim) + 2.3 L COC ha^−1^ + 9.4 L Solution 32 ha^−1^	1×	1×	1×
	Residue	Bromoxynil + 2,4-D	877 g a.i. ha^−1^ + 1097 g a.i. ha^−1^	Deadbolt + 701 g In-Place ha^−1^	1×	1×	1×
2020	Fallow	Glyphosate	1117 g a.e. ha^−1^	GlyStar 5 Extra + 20 g AMS L^−1^	1×	3×	1×
		Bromoxynil + Pyrasulfotole	230 g a.i. ha^−1^ + 40 g a.i. ha^−1^	Huskie + 157 g a.i. ha^−1^ Section 3 (clethodim) + 2.3 L COC ha^−1^ + 9.4 L Solution 32 ha^−1^	1×	3×	1×
	Postharvest	Glyphosate	1117 g a.e. ha^−1^	GlyStar 5 Extra + 20 g AMS L^−1^	1×	3×	1×
		Bromoxynil + Pyrasulfotole	230 g a.i. ha^−1^ + 40 g a.i. ha^−1^	Huskie + 157 g a.i. ha^−1^ Section 3 (clethodim) + 2.3 L COC ha^−1^ + 9.4 L Solution 32 ha^−1^	1×	3×	1×
	Residue	Glyphosate + Bromoxynil + Pyrasulfotole	1117 g a.e. ha^−1^ + 215 g a.i. ha^−1^ + 38 g a.i. ha^−1^	Huskie + GlyStar 5 Extra + 136 g Non-ionic surfactant ha^−1^ + 1.9 L Solution 32 ha^−1^	1×	3×	1×

^a^ Each spray system was calibrated to deliver the recommended herbicide rate according to the label (denoted as 1×). WEED-IT was recalibrated in 2020 to deliver a lower herbicide volume but was unknowingly spraying 3× the intended volume. Consequently, the delivered herbicide rate of WEED-IT in 2020 was 3× higher than uniform and WeedSeeker treatments.

**Table 2:** Pre-spray weed density and cover, and herbicide control efficacy (mean ± SD) for plant density and cover in the fallow trial for glyphosate and bromoxynil + pyrasulfotole herbicides in 2019 and 2020.

			Glyphosate				Bromoxynil + Pyrasulfotole			
Year	Factor	Level	Pre-Spray Density (Plants/m^2^)	Pre-Spray Cover (%)	Density Reduction Efficacy (%)	Cover Reduction Efficacy (%)	Pre-Spray Density (Plants m^2^)	Pre-Spray Cover (%)	Density Reduction Efficacy (%)	Cover Reduction Efficacy (%)
2019	Spray system				***	***			**	**
		Uniform	30.8	23.9	86.6 ± 13 a	68.3 ± 24 a	25.2	25.6	58.1 ± 25 a	34.4 ± 37 a
		WEED-IT	34.4	35.9	73.5 ± 11 b	19.4 ± 27 b	38.0	38.1	38.0 ± 20 b	5.9 ± 13 b
	Weed Community				ns	*			**	ns
		Natural	33.2	22.8	81.6 ± 16	61.2 ± 30 a	23.4	19.0	40.9 ± 26 b	25.4 ± 36
		Kochia	34.6	24.9	83.3 ± 12	44.2 ± 37 ab	32.0	25.2	40.4 ± 26 b	16.5 ± 29
		Russian thistle (RT)	29.8	41.9	75.3 ± 12	26.0 ± 32 b	39.2	51.4	62.9 ± 14 a	18.5 ± 29
	Interaction				***	***			**	ns
		Uniform_Natural	28.4	12.3	83.4 ± 21 ab	78.8 ± 16 a	18.2	12.4	53.7 ± 30 ab	40.0 ± 44
		Uniform_Kochia	39.0	24.0	91.7 ± 7 a	74.5 ± 20 a	32.0	22.2	51.4 ± 25 ab	30.3 ± 36
		Uniform_RT	24.8	35.4	84.7 ± 8 abc	51.2 ± 27 ab	25.2	42.4	69.2 ± 15 a	32.8 ± 36
		WEED-IT_Natural	38.0	33.4	80.0 ± 11 abc	43.7 ± 32 ab	28.4	25.6	28.1 ± 12 b	10.9 ± 20
		WEED-IT_Kochia	30.0	25.9	74.9 ± 9 bc	13.7 ± 19 b	32.0	28.2	29.3 ± 23 b	2.7 ± 6
		WEED-IT_RT	35.0	48.5	65.8 ± 8 c	0.8 ± 2 b	53.2	60.5	56.6 ± 12 ab	4.2 ± 7.8
2020	Spray system				ns	***			**	**
		Uniform	54.6	19.2	43.5 ± 24	23.4 ± 30 a	32.0	8.1	62.9 ± 21 a	9.6 ± 28 a
		WeedSeeker	66.8	14.7	27.6 ± 28	1.7 ± 6 b	31.6	10.9	31.1 ± 28 b	0.0 ± 0 b
		WEED-IT	55.0	8.3	31.4 ± 27	0.0 ± 0 b	39.6	6.9	47.3 ± 30 ab	1.6 ± 8 ab
	Weed Community				ns	ns			ns	ns
		Natural	40.0	12.5	30.3 ± 25	9.2 ± 24	26.6	8.4	46.8 ± 31	6.3 ± 18
		Kochia	51.2	12.3	34.7 ± 25	5.6 ± 14	29.4	9.1	53.9 ± 24	1.0 ± 5
		RT	85.2	17.5	37.5 ± 31	10.2 ± 22	47.2	8.4	40.5 ± 31	3.9 ± 13
	Interaction				ns	**			ns	*
		Uniform_Natural	37.0	17.5	35.7 ± 22	27.7 ± 36 a	22.2	8.3	63.3 ± 27	14.1 ± 28 a
		Uniform_Kochia	45.2	14.8	42.6 ± 22	12.7 ± 22 ab	29.8	6.8	66.6 ± 17	2.9 ± 8 ab
		Uniform_RT	81.6	25.2	52.1 ± 28	29.7 ± 31 a	44.2	9.2	58.7 ± 18	11.8 ± 28 a
		WeedSeeker_Natural	45.0	12.9	22.2 ± 26	0.0 ± 0 b	26.6	10.3	24.5 ± 29	0.0 ± 0 b
		WeedSeeker_Kochia	72.0	15.6	37.7 ± 28	4.0 ± 10 b	24.8	11.1	39.6 ± 21	0.0 ± 0 b
		WeedSeeker_RT	83.6	15.8	22.8 ± 29	1.0 ± 3 b	43.6	11.2	29.3 ± 35	0.0 ± 0 b
		WEED-IT_Natural	36.8	7.0	32.9 ± 28	0.0 ± 0 b	31.2	6.6	52.7 ± 26	4.9 ± 14 ab
		WEED-IT_Kochia	37.8	6.4	23.8 ± 23	0.0 ± 0 b	34.0	9.4	55.7 ± 27	0.0 ± 0 b
		WEED-IT_RT	90.2	11.5	37.5 ± 31	0.0 ± 0 b	53.8	4.8	33.4 ± 34	0.0 ± 0 b

Significance indicated by ns *p* > 0.05, * *p* ≤ 0.05, ** *p* ≤ 0.01, and *** *p* ≤ 0.001 for main effects and letters a, b, and c (α = 0.05) for means separation in each column.

**Table 3:** Pre-spray weed density and cover, and herbicide control efficacy (mean ± SD) for plant density and cover in the postharvest trial for glyphosate and bromoxynil + pyrasulfotole herbicides in 2019 and 2020.

			Glyphosate				Bromoxynil + Pyrasulfotole			
Year	Factors	Level	Pre-Spray Density (Plants/m^2^)	Pre-Spray Cover (%)	Density Reduction Efficacy (%)	Cover Reduction Efficacy (%)	Pre-Spray Density (Plants m^2^)	Pre-Spray Cover (%)	Density Reduction Efficacy (%)	Cover Reduction Efficacy (%)
2019	Spray system				ns	**			**	**
		Uniform	13.4	20.6	46.1 ± 35	50.4 ± 36 a	13.4	22.5	62.0 ± 36 a	74.6 ± 34 a
		WeedSeeker	8.9	15.6	30.2 ± 29	24.1 ± 35 b	13.3	25.2	30.5 ± 31 b	35.9 ± 38 b
		WEED-IT	13.9	19.3	28.9 ± 29	23.9 ± 30 b	13.1	18.0	40.0 ± 38 ab	42.6 ± 40 b
	Weed Community				*	ns			ns	*
		Natural	3.8	7.8	20.9 ± 27 b	29.3 ± 35	5.8	5.6	33.8 ± 37	35.3 ± 42 b
		Kochia	9.3	6.0	43.9 ± 33 a	26.1 ± 34	11.0	12.2	42.8 ± 36	49.8 ± 38 ab
		Russian thistle (RT)	23.0	41.8	40.5 ± 32 ab	43.1 ± 37	22.9	47.9	55.8 ± 35	68.0 ± 37 a
	Interaction				**	*			ns	**
		Uniform_Natural	3.1	3.7	15.5 ± 22 b	34.6 ± 35 ab	5.8	4.4	50.9 ± 48	63.3 ± 45 ab
		Uniform_Kochia	6.8	5.0	54.6 ± 35 ab	45.0 ± 42 ab	10.8	10.6	61.7 ± 33	71.5 ± 36 ab
		Uniform _RT	30.2	53.2	68.2 ± 26 a	71.6 ± 23 a	23.6	52.4	73.4 ± 23	89.0 ± 12 a
		WeedSeeker_Natural	3.4	7.4	33.9 ± 29 ab	30.6 ± 39 ab	5.8	9.4	23.0 ± 28	26.9 ± 39 ab
		WeedSeeker_Kochia	7.8	6.4	34.0 ± 33 ab	15.4 ± 29 b	10.3	13.5	27.6 ± 31	29.7 ± 30 ab
		WeedSeeker_RT	15.3	33.1	22.7 ± 26 ab	26.2 ± 38 ab	23.7	52.7	40.9 ± 33	51.2 ± 43 ab
		WEED-IT_Natural	4.8	12.4	13.2 ± 28 b	22.7 ± 33 ab	5.9	3.0	27.5 ± 30	15.9 ± 29 b
		WEED-IT_Kochia	13.3	6.6	43.1 ± 31 ab	17.8 ± 25 ab	12.1	12.5	39.2 ± 39	48.1 ± 40 ab
		WEED-IT_RT	23.5	39.0	30.5 ± 24 ab	31.4 ± 34 ab	21.3	38.4	53.2 ± 43	63.8 ± 39 ab
2020	Spray system				ns	*			ns	ns
		Uniform	9.8	6.5	12.3 ± 18	34.6 ± 32 a	10.0	4.2	6.6 ± 10	24.6 ± 36
		WeedSeeker	7.9	5.6	5.2 ± 13	15.6 ± 23 b	11.4	4.9	1.5 ± 4	8.5 ± 18
		WEED-IT	6.8	7.1	22.2 ± 31	47.4 ± 39 a	8.1	4.8	3.5 ± 9	15.7 ± 28
	Weed Community				***	***			***	***
		Natural	4.0	3.4	1.3 ± 5 b	20.7 ± 25 b	4.2	1.0	0.3 ± 1.6 b	0.0 ± 0 b
		Kochia	1.7	2.7	1.9 ± 6 b	18.1 ± 28 b	4.8	1.0	0.0 ± 0 b	0.9 ± 4 b
		RT	18.8	13.0	36.5 ± 26 a	58.8 ± 33 a	20.5	11.9	11.2 ± 12 a	47.9 ± 31 a
	Interaction				***	***			***	***
		Uniform_Natural	4.4	4.1	0.0 ± 0 c	21.8 ± 26 bc	4.1	1.0	0.0 ± 0 b	0.0 ± 0 b
		Uniform_Kochia	2.1	2.2	2.5 ± 7 c	12.4 ± 18 bc	6.7	1.1	0.0 ± 0 b	2.7 ± 8 b
		Uniform_RT	23.0	13.4	34.3 ± 12 ab	69.5 ± 14 ab	19.3	10.5	19.7 ± 8 a	71.1 ± 20 a
		WeedSeeker_Natural	5.8	3.8	3.8 ± 8 bc	16.9 ± 25 bc	5.0	1.1	1.0 ± 3 b	0.0 ± 0 b
		WeedSeeker_Kochia	1.6	2.5	0.0 ± 0 c	8.2 ± 16 c	4.3	1.1	0.0 ± 0 b	0.0 ± 0 b
		WeedSeeker_RT	16.2	10.6	11.7 ± 20 bc	21.8 ± 27 bc	24.8	12.6	3.4 ± 7 b	25.4 ± 25 ab
		WEED-IT_Natural	1.8	2.5	0.0 ± 0 c	23.3 ± 27 bc	3.4	0.8	0.0 ± 0 b	0.0 ± 0 b
		WEED-IT_Kochia	1.3	3.6	3.1 ± 9 c	33.7 ± 41 abc	3.5	0.9	0.0 ± 0 b	0.0 ± 0 b
		WEED-IT_RT	17.2	15.1	63.6 ± 11 a	85.2 ± 9 a	17.3	12.6	10.6 ± 14 ab	47.1 ± 30 a

Significance indicated by ns *p* > 0.05, * *p* ≤ 0.05, ** *p* ≤ 0.01, and *** *p* ≤ 0.001 for main effects and letters a, b, and c (α = 0.05) for means separation in each column.

**Table 4:** Pre-spray weed cover and herbicide control efficacy (mean ± SD) for plant cover in the residue trial during 2019 and 2020.

Year	Factor	Level	Pre-Spray Cover (%)	Cover Reduction Efficacy (%)
2019	Spray system			***
		Uniform	12.0	76.8 ± 29 a
		WeedSeeker	13.6	43.2 ± 33 b
		WEED-IT	9.8	21.0 ± 28 b
	Stubble height			ns
		Short	5.7	32.7 ± 40
		Tall	16.1	51.9 ± 35
		Tarp	13.5	56.3 ± 35
	Interaction			***
		Uniform_Short	5.5	64.4 ± 42 ab
		Uniform_Tall	22.4	80.5 ± 20 a
		Uniform_Tarp	8.1	85.4 ± 16 a
		WeedSeeker_Short	7.6	27.5 ± 36 ab
		WeedSeeker_Tall	15.7	46.4 ± 30 ab
		WeedSeeker_Tarp	17.5	55.7 ± 30 ab
		WEED-IT_Short	4.1	6.3 ± 12 b
		WEED-IT_Tall	10.2	28.9 ± 34 ab
		WEED-IT_Tarp	15.1	27.7 ± 31 ab
2020	Spray system			**
		Uniform	46.4	93.8 ± 5 ab
		WeedSeeker	53.7	88.2 ± 10 b
		WEED-IT	47.6	94.9 ± 5 a
	Stubble height			ns
		Short	54.0	94.2 ± 5
		Tall	50.0	91.0 ± 7
		Tarp	43.8	91.8 ± 10
	Interaction			*
		Uniform_Short	45.8	94.2 ± 6 ab
		Uniform_Tall	46.8	91.3 ± 6 abc
		Uniform_Tarp	46.7	95.9 ± 4 a
		WeedSeeker_Short	59.1	92.4 ± 6 abc
		WeedSeeker_Tall	53.9	89.2 ± 8 bc
		WeedSeeker_Tarp	48.5	83.1 ± 13 c
		WEED-IT_Short	57.1	95.9 ± 4 a
		WEED-IT_Tall	49.5	92.4 ± 7 abc
		WEED-IT_Tarp	36.2	96.4 ± 4 a

Significance indicated by ns *p* > 0.05, * *p* ≤ 0.05, ** *p* ≤ 0.01, and *** *p* ≤ 0.001 for main effects and letters a, b, and c (α = 0.05) for means separation.

**Table 5:** Herbicide volume saved by WeedSeeker and WEED-IT in the fallow ^a^, postharvest, and residue trials in 2019 and 2020.

Year	Trial	Sprayer	Continuous Spray Volume (L ha^−1^)	Effective Spray Volume ^b^ (L ha^−1^)	Average Weed Coverage (%)	Area Treated ^c^ (%)	Herbicide Volume Saved (%)
2019	Postharvest	WeedSeeker	192	84	20.4	44	56
		WEED-IT	519	268	18.7	52	48
	Residue	WeedSeeker	192	61	13.6	32	68
		WEED-IT	519	317	9.8	61	39
2020	Fallow	WeedSeeker	192	61	12.8	32	68
	Postharvest	WeedSeeker	192	77	5.3	40	60
		WEED-IT	468	85	6.0	18	82
	Residue	WeedSeeker	192	85	53.7	44	56
		WEED-IT	468	453	47.6	97	3

^a^ Spray volume used by WEED-IT in the fallow 2019 and fallow 2020 trials were not recorded. ^b^ Effective spray volume is the output volume of a spot sprayer in spot spraying mode. ^c^ Area treated is the effective spray volume divided by the continuous spray volume.

## Data Availability

The data presented in this study are available within the article.
